# Influence of insertion sequences on population structure of phytopathogenic bacteria in the *Ralstonia solanacearum* species complex

**DOI:** 10.1099/mic.0.001364

**Published:** 2023-07-17

**Authors:** Samuel T. E. Greenrod, Martina Stoycheva, John Elphinstone, Ville-Petri Friman

**Affiliations:** ^1^​ Department of Biology, University of York, York, UK; ^2^​ Fera Science Ltd, National Agri-Food Innovation Campus, Sand Hutton, York, UK; ^†^​ Present address: Department of Biology, University of Oxford, Oxford, UK; ^‡^​ Present address: Department of Microbiology, University of Helsinki, 00014, Helsinki, Finland

**Keywords:** *Ralstonia solanacearum*, RSSC, insertion sequence, plant pathogenic bacterium, mobile genetic element, diversity

## Abstract

*
Ralstonia solanacearum* species complex (RSSC) is a destructive group of plant pathogenic bacteria and the causative agent of bacterial wilt disease. Experimental studies have attributed RSSC virulence to insertion sequences (IS), transposable genetic elements which can both disrupt and activate host genes. Yet, the global diversity and distribution of RSSC IS are unknown. In this study, IS were bioinformatically identified in a diverse collection of 356 RSSC isolates representing five phylogenetic lineages and their diversity investigated based on genetic distance measures and comparisons with the ISFinder database. IS phylogenetic associations were determined based on their distribution across the RSSC phylogeny. Moreover, IS positions within genomes were characterised and their potential gene disruptions determined based on IS proximity to coding sequences. In total, we found 24732 IS belonging to eleven IS families and 26 IS subgroups with over half of the IS found in the megaplasmid. While IS families were generally widespread across the RSSC phylogeny, IS subgroups showed strong lineage-specific distributions and genetically similar bacterial isolates had similar IS contents. Similar associations with bacterial host genetic background were also observed with IS insertion positions which were highly conserved in closely related bacterial isolates. Finally, IS were found to disrupt genes with predicted functions in virulence, stress tolerance, and metabolism suggesting that they might be adaptive. This study highlights that RSSC insertion sequences track the evolution of their bacterial hosts potentially contributing to both intra- and inter-lineage genetic diversity.

## Data Summary

Raw sequence read data of the RSSC isolates is deposited in the Sequence Read Archive under BioProject accession number PRJNA823737, accessible at https://www.ncbi.nlm.nih.gov/bioproject/PRJNA823737. Sequence accessions are available in Table S1. R code used is available at: https://github.com/SamuelGreenrod/IS_MS. RSSC phylogenetic tree is available at: 10.6084 /m9.figshare.23587770.

**Impact Statement**

This study highlights that insertion sequences are large contributors to genetic diversity in the plant pathogenic *Ralstonia solanacearum* species complex. Further, it suggests that some insertion sequences may be adaptive by disrupting coding sequences and therefore may contribute to variation in pathogen virulence and competitiveness both within and between phylogenetic lineages.

## Introduction

Genetic variation is the raw material for selection and evolutionary change. In bacteria, genetic variation is generated by replication errors resulting in single nucleotide polymorphisms (SNPs), small and large genome rearrangements, and gene gain and loss via horizontal gene transfer [1]. Further modifications that affect gene expression include epigenetic modifications via DNA methylation and gene disruptions caused by the movement of mobile genetic elements including integrated bacteriophages (prophages) and insertion sequences (IS). IS are small transposable elements which can move within a single genome or horizontally between different cells via mobile genetic elements [2], generating within and between population genetic variation. IS have two main structural components: a transposase, the enzyme responsible for IS translocation; and transposase-flanking terminal inverted repeats, linked to transposase binding, DNA cleavage, and strand transfer [3]. IS are highly diverse and are classified into families based on the gene sequence of their transposase, and further into subgroups based on the presence and order of transposase protein domains (for review see [4]). In contrast to transposons, IS seldom carry auxiliary genes that would have effects on host bacterial fitness [4]. Instead, the impact of IS on host fitness is dependent on their genomic location. Most fitness-associated IS insertions are found within coding sequences, resulting in deactivation of particular genes. IS-mediated gene disruptions are prevalent across many bacterial taxa and have been found to alter bacterial traits including antimicrobial resistance [5–7], virulence [8–10], and metabolism [11, 12]. Moreover, IS can also alter host gene expression by inserting close to genes which can result in gene promoter disruptions or even increased neighbouring gene expression as some IS contain either entire or partial promoter regions. Examples leading to differential gene expression caused by IS insertions include increased host resistance to antibiotics [13, 14] and phages [15], and activation of virulence [16] and metabolic pathways [17, 18].

While IS have been studied in both eukaryotic host-associated and free-living bacteria, most of the research on IS host fitness effects has focused on human bacterial pathogens (for review see: [19]) and only a few studies have been published on plant pathogenic bacteria. In the rice pathogen *Xanthomonas oryzaei*, IS have been shown to inactivate genes in the *gum* cluster responsible for the biosynthesis of extracellular polysaccharides [20], in addition to disrupting the virulence-related *purH* gene [21]. Moreover, IS virulence gene disruptions have been found in *Pseudomonas syringae* pv. *phaseolicola*, responsible for halo blight of the common bean, via IS insertion into a potential avirulence gene [22]. Notably, in the same genera, IS have also been linked to the horizontal transfer of virulence genes [23–25], suggesting they likely play a role in both elevated and reduced host virulence. Recently, IS content was also investigated in the plant pathogenic bacterial *Ralstonia solanacearum* species complex (RSSC), a causative agent of bacterial wilt disease, through a genomic analysis of 62 complete RSSC genome sequences in the NCBI database [26]. This study identified 20 IS families, including some which were widespread across the bacterial phylogeny and others which were only found in specific host phylogenetic lineages. IS were often located nearby, or inserted into, genes with potential roles in virulence, resistance to oxidative stress, and toxin production, potentially affecting the fitness of their hosts [26]. These findings supported previous analyses of RSSC IS which identified disruptions in type III effectors [27] and in the global virulence regulator phcA [28], the latter of which resulted in spontaneous phenotypic conversion between non-virulent and virulent pathogen genotypes. It was also recently shown that RSSC IS are highly mobile under lab conditions and may contribute to host competitiveness and environmental stress tolerance [29]. However, thus far our understanding of IS in RSSC is based on experimental studies of individual isolates [28, 29] and a small number of genomes derived from publicly available databases [30]. As a result, the wider diversity and distribution of IS in RSSC is unknown. In addition, we poorly understand to what extent IS-driven variation follows the host phylogeny, concealing whether IS generally transmit vertically or if they are more often characterised by horizontal movement between lineages.

RSSC strains have broad host ranges infecting over 200 plant species within at least 50 families [31, 32]. They contain a bi-partite genome comprised of a chromosome and an immobile megaplasmid, and are genetically diverse, being classified into four lineages, termed phylotypes [33]. Phylotypes generally follow their geographical origin: phylotype I includes strains originating primarily from Asia, phylotype II from America, phylotype III from Africa and surrounding islands in the Indian ocean, and phylotype IV from Indonesia, Japan, and Australia [34]. The four phylotypes have more recently been redefined as three separate species, including *R. solanacearum sensu stricto* (phylotype II), *R. pseudosolanacearum* (phylotypes I and III) and an array of *R. syzygii* subspecies (phylotype IV) [35]. Considerable variation exists between and within RSSC phylotypes regarding their metabolic versatility [36], tolerance to environmental stresses including starvation and low temperatures [37, 38], and disease severity [39]. This has been linked to a diverse accessory genome [40, 41] which includes mobile genetic elements such as prophages [30, 42]. A recent analysis of RSSC prophages found that, while prophages were highly diverse, they were generally bacterial host phylotype-specific [42]. In addition, prophage content tightly followed the host phylogeny with genetically similar hosts containing similar prophages. Some IS families have been reported to also have lineage-specific distributions [26] being exclusively found in specific RSSC species. Therefore, they may have similar distribution patterns to prophages and have limited horizontal transfer between compared to within bacterial lineages. Addressing this hypothesis requires a deeper exploration of the relationship between IS content and the host phylogeny and an assessment of potential IS fitness effects across the RSSC.

The large diversity of the strains within the complex and the economic losses associated with the disease make the RSSC a salient target for IS content analysis. While a recent study made a significant contribution to understanding RSSC IS using publicly available RSSC genomes [26], it had a sampling bias with low representation of strains from phylotypes IIA and IIB, missing a subset of hosts which are cold-adapted [37]. In this study, IS were identified in a new representative collection of 356 RSSC isolates. These included isolates from all four phylotypes and six continents, with extensive sampling of phylotypes I and IIB. We specifically aimed to: i) characterise the total diversity and distribution of IS in RSSC; ii) assess the relationship between IS content and the host phylogeny; iii) determine the distribution of IS positions within RSSC genomes; and iv) investigate the potential impact of IS movement on host fitness-associated genes. IS were initially identified in 27 Nanopore-assembled and five complete reference genomes with ISEScan [43] to determine IS diversity. Representative IS were then used to identify IS in all isolates using short read data with ISMapper [44]. IS distributions were characterised by assessing lineage-specificity of both IS content and IS genome positions, with the relationship between IS content and host genetic background determined by comparing IS Bray-Curtis and host genetic dissimilarities. Finally, potential IS fitness effects were investigated based on their proximity to neighbouring genes.

## Methods

### RSSC hosts, sequencing, and genome assembly

A total of 356 RSSC Illumina short-read genome sequences were extracted from a previous publication on RSSC genome diversity (see [42] for information on genomic DNA extraction, sequencing, and genome assembly). Of these, 27 isolates were chosen for additional long read re-sequencing with Oxford Nanopore MinION which was performed by the technological facility at the University of York. Guppy (https://nanoporetech.com/) was used for basecalling and hybrid assemblies were produced using the Unicycler (v0.4.8) pipeline on strict mode [45] (Table S1, available in the online version of this article). After assembly, some small contigs were filtered out based on sequence similarity and size. To classify the genomes, a pangenome analysis was performed on 329 high quality short-read and 27 hybrid genome assemblies and *R. picketti* 12b was used as an outgroup. This was used to generate a maximum likelihood phylogenetic tree and genomes were assigned to phylotypes based on clustering with known phylotypes included in the tree (see [42] for information on tree generation and phylotype assignment).

### IS detection in Nanopore and reference genomes

IS were detected in 27 Nanopore-assembled genomes (Table S1) and five complete reference genomes (GMI1000, phylotype I; K60, phylotype IIA; UY031, phylotype IIB; CMR15, phylotype III; PSI07, phylotype IV), representing different phylotype lineages in the RSSC phylogeny. Firstly, putative full-length IS were identified with ISEScan v.1.7.2.3 [43] using the removeShortIS parameter to remove partial IS copies. In total, 2768 full length IS were identified. These were filtered by removing IS duplicates based on whether they belonged to the same IS family and had the same length or if they had identical terminal inverted repeats. After filtering, 861 IS were retained and used for subsequent analyses. IS diversity was then assessed by determining IS sequence similarity using Mash v2.2 [46] and generating a pairwise Mash distance matrix using the ‘mash triangle’ function with sketch size=10 000. IS were clustered based on sequence similarity using K-means clustering with the R package ‘pheatmap’ v1.0.12. The optimal number of clusters was determined using a Silhouette plot with the R package ‘factoextra’ v1.0.7. A total of 66 IS clusters were identified and one IS representative was selected at random from each cluster. To determine IS identities, IS representatives were blasted against the ISFinder database (https://isfinder.biotoul.fr/) with successful hits determined using an E-value <E^−50^ threshold. For successful hits, the ISFinder database copy of the IS was downloaded. Notably, successful hits were found for 41/66 (62 %) clusters suggesting some IS diversity may have been missed.

### IS detection in RSSC isolates from short read data

IS were identified in all 356 RSSC isolates using only short read data with ISMapper v.2.0.2 [44]. Briefly, ISMapper maps short read data to reference IS, identifying reads that map to and overhang the 3′ and 5′ IS flanks. Mapped reads are then further mapped to an annotated reference bacterial genome and IS positions are determined where 3′ and 5′ flanking reads both map to similar genomic locations. Representative IS sequences downloaded from the ISFinder database were used as references. As gene content varies between RSSC lineages, different reference bacterial genomes were used depending on the phylotype of the isolate being analysed. As a result, depending on their phylotype classification, reads from isolates were mapped to GMI1000, K60, UY031, CMR15, and PSI07 strains. Annotated bacterial genomes (GenBank format file, gbff) were downloaded from the NCBI database (Table S2). After running ISMapper, IS hits were filtered to remove potential false positives. IS with unknown 5′ or 3′ coordinates were removed. Further, IS hits that overlapped within the same isolates were de-duplicated as they likely represent different reference IS mapping to the same location. As all overlaps occurred with IS from the same family, the remaining de-duplicated IS were given an ‘Unknown_’ + IS family label (e.g Unknown_IS5). Finally, IS that were found to disrupt transposases, likely representing intra-IS insertions, were removed. This is because IS that map inside multi-copy genes will map to all copies irrespective of the true IS content and therefore may generate spurious hits [44]. To see whether IS detection with short read data has lower accuracy than with long read assemblies, IS copy number from each method was compared using the 27 Nanopore-sequenced isolates (Table S3). While there was a significant correlation in IS copy number when using short and long read data (Kendall’s *P*<0.01; Fig. S2A), short read data had lower IS detection power, identifying on average only ~73 % of the IS found with long read assemblies. Therefore, to fairly compare IS between all isolates, IS were only detected with short read data. Moreover, as read depth can affect IS detection power, the average per base read depth for each isolate was determined and compared between host phylotypes (Fig. S2B). Read depth was calculated by first aligning paired-end reads to the isolate chromosome or megaplasmid using the Burrows-Wheeler aligner [47] ‘bwa mem’ command. Per base read depth was then determined using SAMtools [48] ‘sort’ and ‘depth’ commands, including bases with no coverage. Although phylotype IIB isolates had significantly greater read depth than phylotype I (Kruskal-Wallis: x^2^=75.1; d.f=4, *P*<0.01), there was no significant difference in read depth for all other pairwise comparisons. Further, as ~99 % of isolates from each phylotype (except for IIA with 93 %) had an average per base read depth >30 x (average read depth: chromosome=67.9 x ± 17 s.d; megaplasmid=59.9 x ± 14.2 s.d) which is above the suggested threshold for correct IS detection [44], read depth likely had a minimal impact on IS detection and copy number.

### Determining the relationship between IS content, position and host genetic background

The relationship between IS content, position, and host genetic background was first assessed by comparing IS profiles between host phylotypes using principal coordinate analysis. Differences in IS subgroup content between isolates were determined by calculating IS Bray-Curtis dissimilarities with the R package ‘vegan’ v.2.5–7, which accounts for presence, absence, and relative abundance of IS subgroups in host genomes. Principal coordinate analysis of Bray-Curtis dissimilarities was conducted using the R package ‘ape’ v.5.6–1 [49] and IS content differences between phylotypes were tested statistically using ANOSIM with 9999 permutations.

IS content and bacterial host genetic background was further compared by calculating the congruence between the RSSC phylogeny and a UPGMA tree constructed using IS Bray-Curtis dissimilarities (previously described in [42]). Briefly, a IS Bray-Curtis UPGMA tree was constructed from a pairwise Bray-Curtis dissimilarity matrix and a tanglegram was generated between the RSSC ML tree and the IS Bray-Curtis UPGMA tree. IS positions and host genetic background were investigated using a similar approach by calculating congruence between the RSSC phylogeny and a UPGMA tree constructed using IS position Jaccard index similarities. Congruence was only calculated within phylotypes as IS positions were determined using genomes of different reference strains between phylotypes. Congruence between the RSSC ML tree and the IS Bray-Curtis/Jaccard UPGMA trees was assessed using Procrustes Approach to Cophylogenetic Analysis (PACo) v.0.4.2 [50] in R.

### Data visualisation and statistical analysis

Statistical analyses and data visualisation were carried out using Microsoft Excel v.2102, R v.4.0.3 and RStudio v1.4.1103. The difference in IS copy number between short read IS detection (ISMapper) and long read detection (ISEScan) was determined using a Kendall-rank correlation. Read depth and IS copy number was compared between phylotypes using Kruskal-Wallis tests followed by Dunn’s post-hoc test. IS copy number was compared between chromosome and megaplasmid using a Wilcoxon signed-rank test. The difference in IS copy number and the number of IS close to (< 100 bp from start codon) or inside genes in the chromosome and megaplasmid were tested using non-parametric paired Wilcoxon signed-rank tests. Graphs and heatmaps were made using the R package ‘ggplot2’ v.3.3.3. The Bray-Curtis UPGMA and ML phylogenetic trees were visualised using the R ‘ggtree’ package v.2.1.4 [51].

## Results

### Insertion sequence abundance and distribution across *Ralstonia solanacearum* species complex

**Fig. 1.**
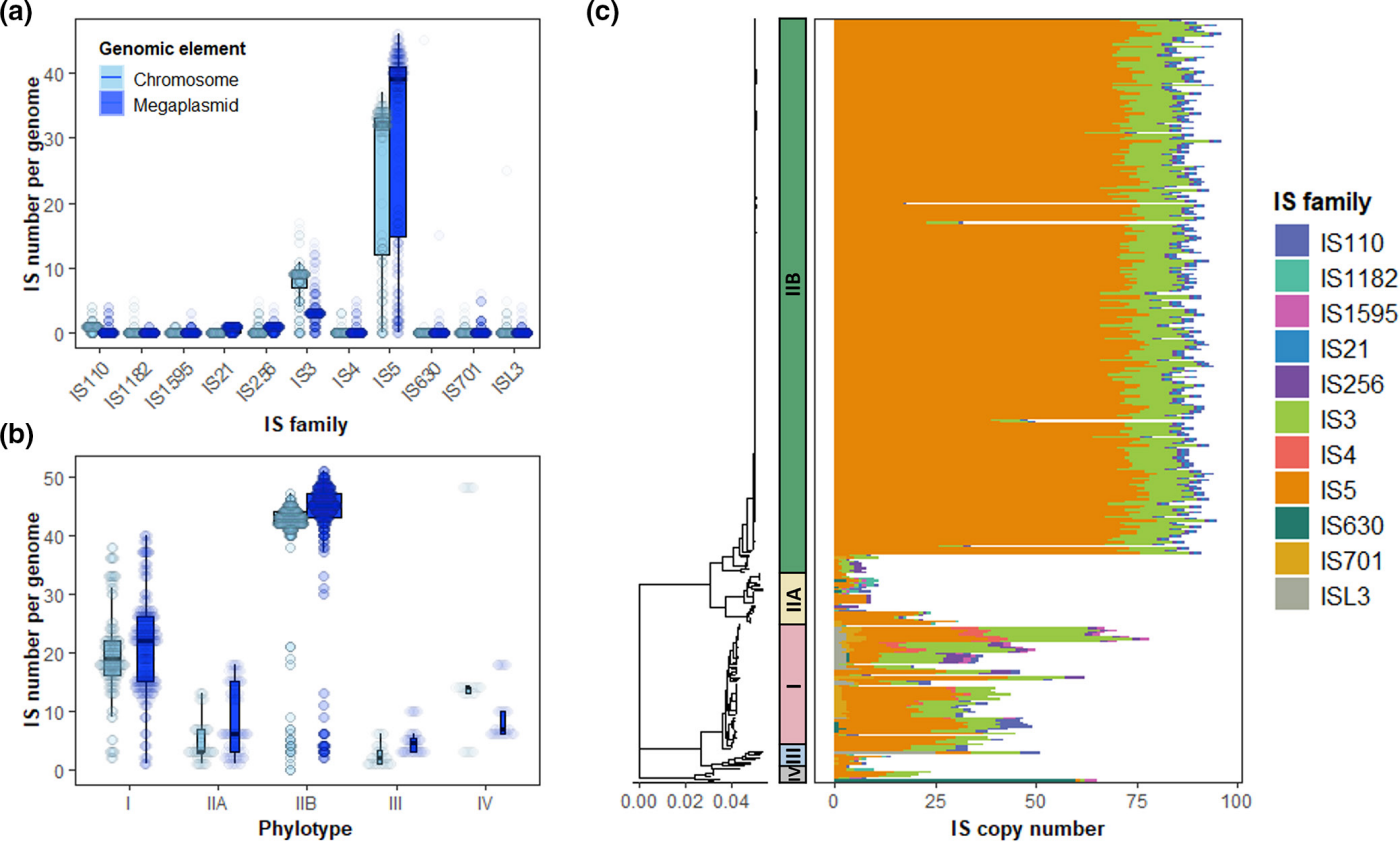
IS number and content varies across the RSSC phylogeny. (**a**) Boxplots of IS prevalence for each IS family for both the chromosome and megaplasmid. (**b**) Boxplots showing the number of IS in isolates from each phylotype in the phylogeny. For both a and b, IS prevalence in both the chromosome (light blue) and the megaplasmid (dark blue) is shown. (**c**) Left side shows RSSC phylogeny with coloured bars showing phylotype label; right side presents a heatmap of IS prevalence coloured by IS family. Scale bar (nucleotide substitutions per site) is shown below phylogeny.

We first compared insertion sequence content and distribution across the genomes of 356 RSSC bacterial isolates. All bacterial isolates contained IS and a total of 24 732 IS were identified (Table S4). A significantly higher number of IS were identified in the megaplasmid (12734 IS) than in the chromosome (11 998 IS; Wilcoxon: V=43394, *n*=356, *P*-value<0.001; Fig. S3) despite the megaplasmid being approximately half the size of the chromosome [33]. Insertion sequences belonged to eleven IS families of which the most prevalent included IS5 (78.9 %) and IS3 (15.4 %). The remaining families each comprised less than 2 % of the total number of IS (Fig. 1a). However, greater IS prevalence in the megaplasmid was not consistent across all IS families and IS from IS110 and IS3 families were predominantly detected in the chromosome. IS copy number was significantly different between phylotypes overall (Kruskal-Wallis: x^2^=151.1; d.f=4, *P*<0.001). However, this result was driven by uneven representation of isolates across phylotypes I (59), IIA (15), IIB (269), III(8), and IV (5), which resulted in overrepresentation of two insertion sequences that were common to phylotype IIB bacterial isolates: IS5 and IS3 (Fig. 1c). Nonetheless, IS5 and IS3 were highly prevalent across the RSSC phylogeny despite the oversampling of phylotype IIB isolates (Fig. 1b, c). In addition to IS5 and IS3, certain lower abundance IS families, such as IS110 and IS256, were also present across the phylogeny. However, a small number of families were found in specific phylotypes. For example, phylotype I isolates almost uniquely contained ISL3 and IS4 and most of the IS701 (89.6 %) and IS1595 (72 %) IS. Phylotype IIB exclusively contained IS21 IS. Therefore, whilst RSSC IS family content was dominated by IS5 and IS3, lineage-specific presence-absence patterns were observed across the phylogeny.

### Insertion sequence content tracks host phylogeny at IS subgroup level

**Fig. 2.**
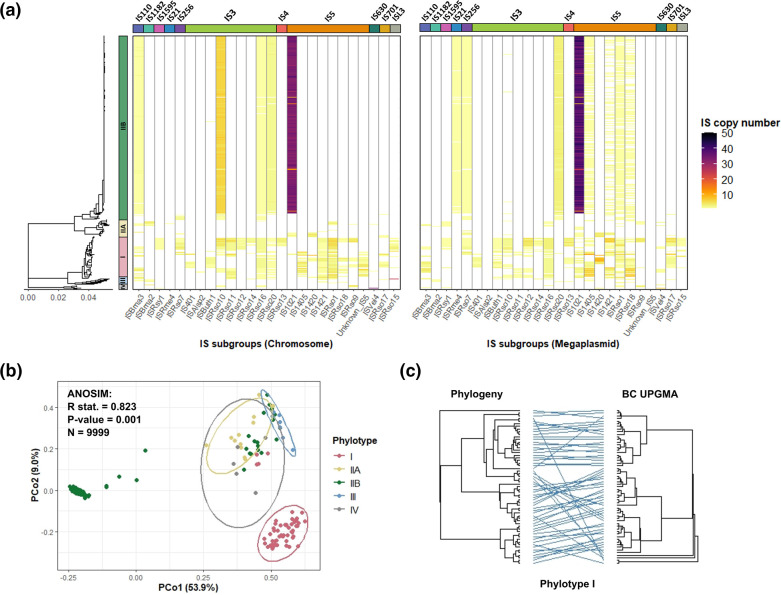
IS subgroups generally show host lineage-specificity and widespread IS are found in different genomic elements depending on host phylotype. (**a**) Heatmap of IS subgroup prevalence across the RSSC phylogeny separated by genomic element (chromosome on the left and megaplasmid on the right). IS subgroups are clustered by IS family and shown with coloured bars above the heatmaps. Scale bar (nucleotide substitutions per site) is shown below. (**b**) PCoA plot based on pairwise isolate IS content Bray-Curtis dissimilarities. Points are coloured by host phylotypes and ellipses show predicted phylotype distributions around the centroid. Phylotype IIB does not show an ellipse due to bimodal distribution caused by clonal and non-clonal sub-lineages. Top-left shows the results of ANOSIM including the R statistic (measure of phylotype IS distinguishability), *P*-value, and number of permutations. (**c**) Tanglegram representing the analysis of congruence between phylotype I isolate phylogeny (left side) and UPGMA tree (right side) calculated using Bray-Curtis dissimilarity of IS presence.

IS family distributions were further investigated by looking at the distributions of intra-family IS subgroups in the chromosome and megaplasmid (Fig. 2a). While the highly prevalent IS5 and IS3 families were found to contain eight and nine IS subgroups, respectively, only individual IS subgroups were identified for other IS families. In contrast to IS5 and IS3 families that were generally widespread and found in all phylotypes, IS5 and IS3 subgroups showed lineage-specific distributions. The IS5 subgroup IS1021 comprised most of the IS5 IS in the large clonal IIB sub-lineage in both the chromosome and megaplasmid and was present only in low abundance in other phylotypes. The remaining IS5 subgroups were primarily found in phylotype I isolates, although some were also found in phylotypes IIA and IV. Similarly, IS3 subgroups in the clonal IIB sub-lineage primarily included ISRso20 which was present in both chromosome and megaplasmid and ISRso10 which was only found in the chromosome. The remaining IS3 subgroups were mainly found in phylotype I. IS subgroup lineage-specificity was further verified using a principle-coordinate analysis based on host IS subgroup Bray-Curtis dissimilarities (Fig. 2b). Phylotypes could be significantly distinguished based on their IS contents (ANOSIM: *P*<0.001 for all phylotypes), with particularly strong clustering between the clonal IIB sub-lineage and phylotype I isolates. Notably, a more diverse cluster was also observed containing isolates from all phylotypes, including non-clonal IIB isolates and all IIA, III, and IV isolates. Although overlaps were small with phylotype III isolates, the general clustering between these isolates suggests that their IS contents were similar. Together, these findings suggest that intra-family IS subgroups were mainly phylotype-specific.

To test if the observed host lineage-specificity of IS subgroups could be explained by host genetic similarity, we measured the congruence between the RSSC phylogeny and a UPGMA tree constructed based on IS subgroup Bray-Curtis dissimilarities (Figs 2c and S4). Significant congruence was detected between the total RSSC phylogeny and Bray-Curtis UPGMA tree (M^2^_xy_ = 0.34, *P*<0.001, *N*=1000). To ensure this wasn’t biased by the large clonal IIB sub-lineage the analysis was repeated for each lineage independently including phylotypes III and IV which had relatively lower sampling sizes. Except for the small sampling size phylotype III, significant congruence was found when analysing phylotypes independently (phylotype I - M^2^_xy_ < 0.001, *P*<0.001, *N*=1000; IIA - M^2^_xy_ < 0.001, *P*<0.001, *N*=1000; IIB - M^2^_xy_ = 0.005, *P*<0.001, *N*=1000; III - M^2^_xy_ < 0.001, *P*=0.052, *N*=1000; IV - M^2^_xy_ < 0.001, *P*<0.05, *N*=100) and each lineage-level congruence comparison had improved goodness-of-fit compared to the congruence analysed at the level of the whole isolate collection. This analysis therefore confirms that genetically similar hosts contain similar IS contents.

### Insertion sequence subgroups are found in different genomic elements in different host lineages

While generally being lineage-specific, IS subgroups were often found in low abundance in other phylotypes indicative of recent horizontal IS gain events rather than vertical transmission from a common ancestor. This was investigated further by comparing the prevalence of IS in different genomic elements, namely the chromosome and megaplasmid, between phylotypes (Fig. S5). Some IS subgroups were found in the same genomic element in all phylotypes. For example, the IS3 subgroup ISRso10 was mainly inserted into the chromosome, and the IS5 subgroups IS1021, IS1405, IS1421, and ISRso18 were primarily found in the megaplasmid. However, 42 % of IS subgroups (11/26) had different genomic locations that depended on the host phylotype. For example, the IS1182 subgroup ISBma2 was found in the chromosome in phylotype IIB and III isolates but was only found in the megaplasmid in phylotype IIA. In addition, IS subgroup associations with genomic elements were not conserved within IS families; in phylotype IIB isolates, the IS3 subgroups ISRso10 and ISRso20 were found in the chromosome and megaplasmid, respectively. In contrast, especially in phylotype IIB, IS5 subgroups were primarily found in the megaplasmid. These results suggest that, irrespective of their family background, IS subgroups are found in different genomic elements within different RSSC phylotypes.

### Insertion sequence genomic distribution varies between RSSC phylotypes

**Fig. 3.**
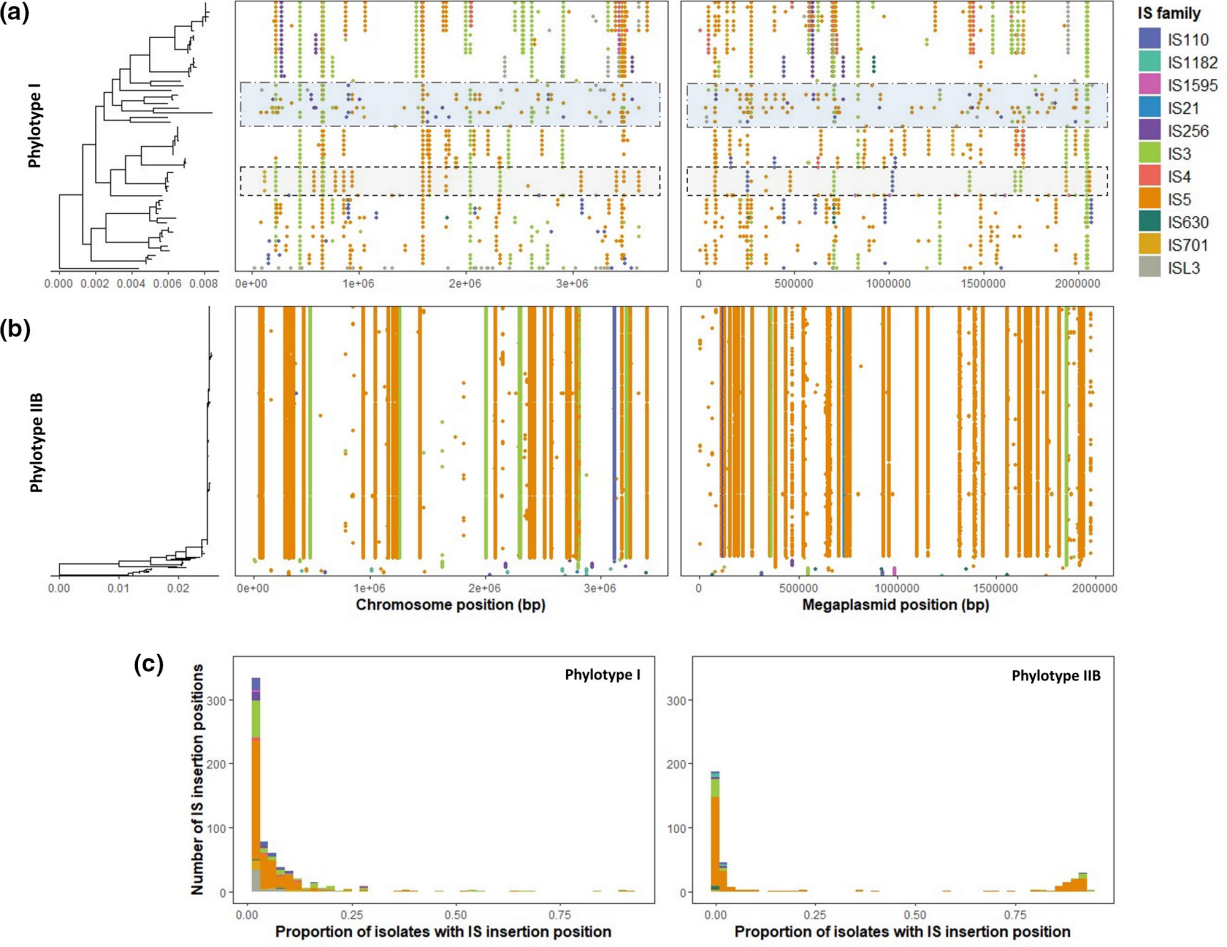
IS locations are highly variable in RSSC genomes but show strong association with RSSC phylogenetic similarity. Dot plots showing the distribution of IS in both the chromosome and megaplasmid across phylogenies of phylotype I (**a**) and phylotype IIB (**b**) isolates. Each dot represents a separate IS position, which are coloured by IS family. Dotted, shaded boxes in (**a**) highlight clades where IS positions are highly variable (top blue box) or highly conserved (bottom grey box) between isolates. Scale bar (nucleotide substitutions per site) is shown below phylogenies. (**c**) Histograms showing the prevalence of IS insertion positions in phylotype I and phylotype IIB isolates. Bars are coloured by IS family.

The potential contribution of IS to RSSC genetic diversity was further investigated by comparing IS insertion positions in the chromosome and megaplasmid within phylotypes (Fig. 3a, b). While IS were found to be broadly distributed in the chromosome and megaplasmid across all RSSC phylotypes, regions with high and low IS densities were observed. In addition, IS positions appeared to be conserved between genetically similar isolates within phylotypes and groups with low IS position conservation were found to have high overall genetic diversity (Fig. 3a). This was investigated further by comparing the congruence between the RSSC phylogeny and phylotype-specific UPGMA trees constructed based on IS position Jaccard distances. Significant congruence was detected between the RSSC phylogeny and Jaccard distance UPGMA trees for phylotype I, IIA, and IIB (phylotype I - M^2^_xy_ <0.001, *P*<0.001, *N*=1000; IIA - M^2^_xy_ <0.001, *P*<0.001, *N*=1000; IIB - M^2^_xy_ = 0.009, *P*<0.001, *N*=1000). This confirms that genetically similar hosts within phylotypes have similar IS positions. Most phylotype I IS positions (76.7 %) were rare being found in <10 % of isolates indicative of recent insertions (Fig. 3c). Similar trends were also observed in phylotype IIA isolates which only showed a few conserved IS positions within phylotype sub-lineages (Fig. S6). In contrast, IS positions in phylotype IIB, primarily containing IS from IS3 and IS5 families, were generally conserved across all isolates indicative of vertical transmission from a common ancestor (Fig. 3c). While this was mainly driven by the over-representation of the clonal phylotype IIB sub-lineage which had low genetic diversity, more diverse phylotype IIA strains had higher variation in IS positions. Overall, IS movement appeared to vary between RSSC lineages depending on their genetic diversity with phylotype I having the highest and phylotype IIB the lowest variability in IS genomic locations.

### Insertion sequences potentially disrupt genes associated with virulence and competitiveness and may contribute to inter-phylotype trait variation

In the RSSC, IS insertions have been shown to occur near to or inside of type III effectors and global virulence regulators affecting host virulence and phenotypic plasticity [28, 30]. Therefore, the proximity of IS to neighbouring genes was investigated. The distance of IS to their closest neighbouring gene was found to have a roughly exponential distribution with most IS being physically close to coding sequences (Fig. S7). Indeed, 71.4 % of all IS identified were found less than 100 bp from a neighbouring gene’s start codon including 78.7 % of chromosomal IS and 66.4 % of megaplasmid IS. Although coding sequences comprise approximately 86.8 % of the RSSC genome [33], only 4.9 % of IS were predicted to disrupt genes (7.1 % of chromosomal IS and 2.8 % of megaplasmid IS) potentially changing their function. Despite IS being significantly more prevalent in the megaplasmid than the chromosome, isolates contained significantly fewer gene-proximate (closer than <100 bp to a start codon) and intra-genic IS in the megaplasmid than in the chromosome (Wilcoxon: V=6842.5, *n*=356, *P*<0.001, Fig. S8).

**Fig. 4.**
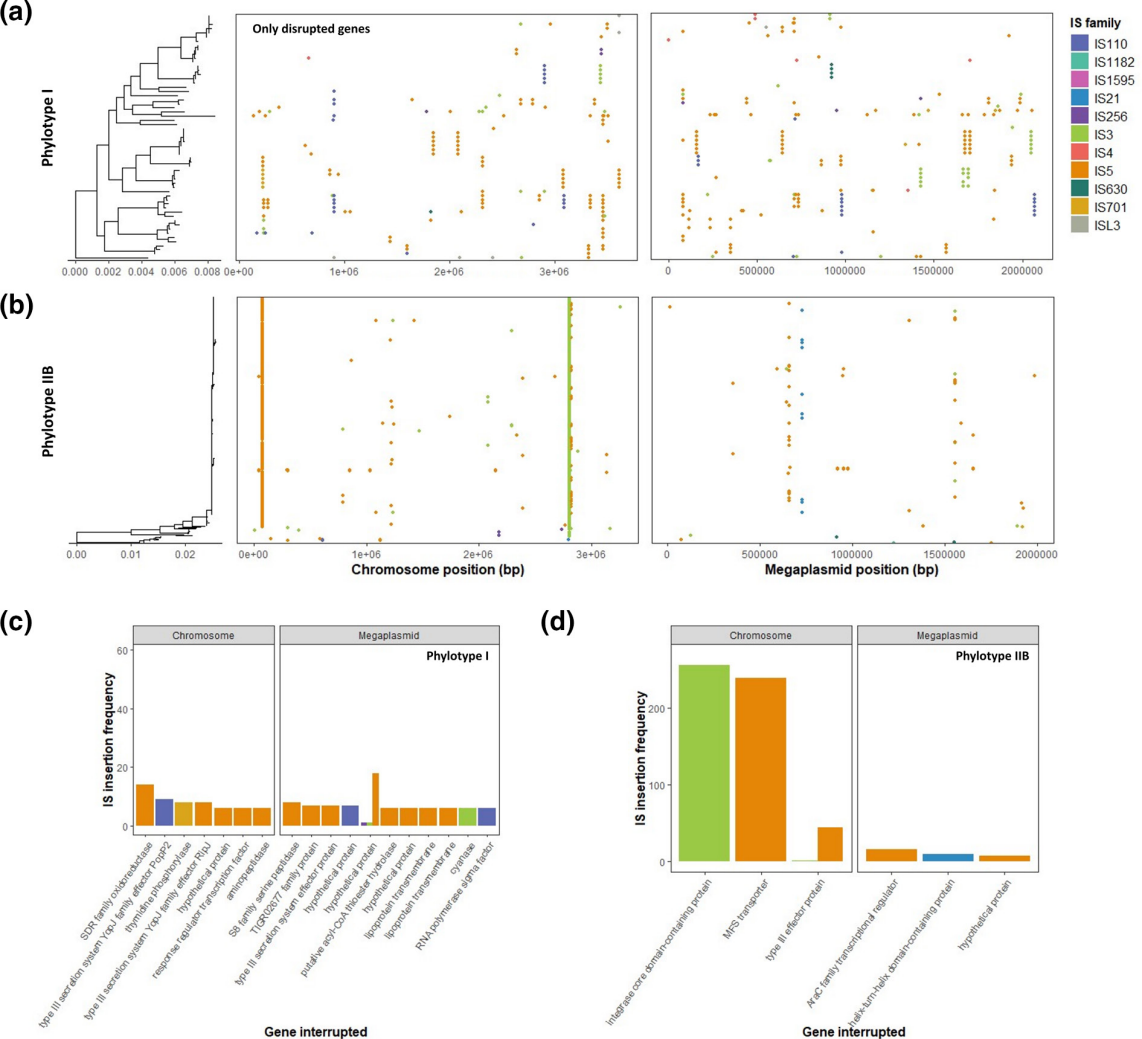
IS disrupt genes across the phylogeny in a phylotype-specific manner. (a and b) Dot plots similar to Fig. 3 showing the distribution of IS gene disruptions in both the chromosome and megaplasmid across phylogenies of phylotype I (**a**) and phylotype IIB (**b**) isolates. Scale bars (nucleotide substitutions per site) are shown below phylogenies. (c and d) Bar plots showing the prevalence and functional annotations of unique disrupted genes in phylotype I (**c**) and phylotype IIB (**d**) isolates. Only genes that contained disruptions in five or more isolates are shown. Genes are ordered by frequency of IS disruption from left to right. Dot plots and bar plots are coloured by IS family.

In total, IS were found to disrupt 336 separate genes across all isolates, including 172 chromosomal genes and 164 megaplasmid genes. As observed with IS genomic positions, gene disruptions were generally conserved within phylotype sub-lineages, indicating they may be propagating via vertical transmission from a common ancestor (Fig. 4a, b). Unique genes that were disrupted by IS in more than five isolates were analysed further (Fig. 4c, d). Of these, 29 disrupted genes were identified and potentially associated with RSSC fitness including bacterial virulence, antibiotic and oxidative stress tolerance, protein modification, cellular metabolism, and RNA-binding. Most gene disruptions (23/29) occurred in phylotype I isolates and had low frequency reflective of the high overall IS activity and genetic diversity in phylotype I. In addition, phylotype I IS gene disruptions were often caused by multiple IS subgroups in different isolates, suggesting they may represent parallel insertions (Fig. S9). In contrast, 2/6 (33 %) IS gene disruptions in phylotype IIB were found in ~90 % of isolates and so were highly conserved. Phylotype IIB gene disruptions were also typically caused by single IS subgroups. This suggests that relatively higher IS movement in phylotype I isolates may contribute to phenotypic variation within this RSSC phylotype.

## Discussion

Insertion sequences contribute to genetic diversity in bacterial pathogens affecting a myriad of traits including metabolism [11, 52], virulence [8, 9, 53], and stress tolerance [5, 7]. In this study, we analysed insertion sequence content in the plant pathogenic RSSC bacterium using a diverse, global collection of 356 RSSC isolates. IS identified in all isolates belonged to eleven IS families, the most prevalent being from the IS5 and IS3 families. Although IS families tended to be widespread, IS subgroups were host phylotype-specific and genetically similar hosts had similar IS contents. Similar patterns were observed with IS insertion positions which were similar in closely related RSSC isolates. IS subgroups were rarely found in multiple phylotypes and, when found, were inserted into different genomic elements with different phylotypes indicative of potential horizontal movement between phylotypes. IS were generally close to neighbouring genes and caused disruptions in several genes potentially associated with bacterial virulence and stress tolerance. Overall, our results suggest that IS elements might be evolving in tandem with their hosts and are potentially contributing to the phenotypic diversity and fitness of the RSSC.

A recent analysis of RSSC insertion sequences provided important insights into the potential diversity and distribution of IS across the RSSC [26]. Here we built on this analysis by including a more representative sampling of new RSSC genomes, including isolates from IIA and IIB phylotype groups that were underrepresented in the previous study. Overall, our results support the previous findings [26]. IS were identified in all isolates analysed although IS copy number varied across the RSSC. We found that a large clonal phylotype IIB sub-lineage contained the greatest number of IS followed by phylotype I and IV, with IIA, III, and non-clonal IIB isolates containing very few IS. Excluding the clonal IIB sub-lineage, our results support previous work suggesting phylotype I has the highest IS copy number [26]. Further, the most prevalent IS were from the IS5 and IS3 families which were widespread across the RSSC with high copy number in the clonal IIB sub-lineage and in phylotype I isolates. Therefore, these IS families are likely the dominant IS families in the RSSC. In contrast with previous findings, non-clonal IIB isolates had very low IS copy number and across all phylotypes we detected fewer IS copies per isolate than were found previously. This is likely due to methodological differences as, while Goncalves *et al*. [26] identified IS in complete genome sequences, we identified IS with short read data which had lower IS detection power than with long read assemblies. This could be because IS detection with short reads depends on reference IS identified from long read assemblies and we identified fewer reference IS than were found previously. In addition, we filtered out intra-IS insertions to avoid spurious hits which, depending on the prevalence of intra-IS insertions across the RSSC, may have resulted in under-estimated IS copy numbers.

Consistent with the findings of Goncalves *et al*. [26], we found that most IS families (6/11) that were less abundant were also less widespread and were mainly found in specific lineages. However, an important extension in our analysis included assessing the distribution of intra-family IS subgroups enabling the detection of nuanced patterns that sometimes occur within IS families [54]. We found that widespread IS5 and IS3 families contain many IS subgroups which have strong lineage-specific distributions and host phylotypes were significantly distinguishable based on IS subgroup content alone. Consequently, IS subgroups appear to be largely restricted within distinct host lineages. Differential IS family copy numbers between lineages have previously been detected in other pathogens [55] and observed to rise in long-term experimental studies [56]. Whilst it is unclear whether these IS families included single or multiple IS subgroups, our results suggest that IS subgroups should be considered in future analyses as their distributions may differ between lineages that contain similar IS family copy numbers. The relationship between IS content and host genetic background was further analysed by calculating the congruence between the host phylogenetic tree and a UPGMA tree constructed based on IS subgroup Bray-Curtis dissimilarities. We found that there was significant congruence between the trees overall and independently within phylotypes, suggesting that genetically similar hosts contain similar IS. These findings mirror those of a recent study conducted on RSSC prophages [42] which found similar lineage-specific distributions and detected significant congruence between prophage content and the host phylogenetic tree. Therefore, multiple mobile genetic elements in RSSC appear to track the evolution of their hosts and act as sources of genetic diversity between lineages.

In contrast with previous analyses [26], we found significantly more IS in the megaplasmid than the chromosome. This was surprising given the RSSC megaplasmid is approximately half the size of the chromosome [33]. One potential explanation is that, although the chromosome and megaplasmid have a similar number of coding sequences relative to their size, over 90 % of the RSSC core genome is found in the chromosome [33, 57]. This includes most house-keeping genes [33] whose disruption would likely reduce host fitness. Therefore, given the megaplasmid mainly contains accessory genes with non-essential functions it may be under weaker purifying selection against IS insertions and so undergoes greater IS propagation. Notably, greater megaplasmid IS prevalence was not found for all IS families and the IS110 and IS3 families were mainly found in the chromosome likely due to their chromosomal prevalence in the large clonal IIB sub-lineage. Although many IS have little to no target specificity [58], IS distributions were inconsistent with random insertions into the chromosome and megaplasmid; if IS insertions were random then the megaplasmid should have proportionately fewer insertions compared to the chromosome in all phylotypes due to its smaller size [33], which was not the case. This could reflect lineage-specific purifying selection against IS inserting into specific genomic elements. Alternatively, some IS are highly specific and target sites of DNA replication [59, 60], motifs upstream of promoters [61, 62], and secondary DNA structures [63–65]. Therefore, IS may have had target preferences in the chromosome or megaplasmid within each phylotype if insertion targets vary phylogenetically. These hypotheses should be addressed in future studies through analyses of RSSC IS target sites and IS fitness effects.

The distribution of IS between the chromosome and megaplasmid was further investigated by comparing the prevalence of IS subgroups in each genomic element (chromosome vs megaplasmid) between phylotypes. Interestingly, although some IS subgroups inserted into the same genomic element across the RSSC, most subgroups inserted into different elements depending on the host phylotype. As IS subgroups tended to have high abundances in specific phylotypes and only low abundances in others, genome region-specificity may have arisen through inter-lineage horizontal IS transfer. IS movement between bacterial lineages has previously been attributed to plasmids [2, 66], integrative and conjugative elements [67], and, rarely, prophages [66]. Both integrative and conjugative elements and prophages have both been found to be lineage-specific [41, 42] and therefore may have limited movement between lineages. Therefore, while the mechanism of IS movement is unclear, IS horizontal transmission may be more likely to have happened via plasmids although additional analyses are required to investigate this.

IS typically contribute to bacterial genetic diversity by either excising or copying themselves and inserting into different parts of the genome [4]. To determine whether IS movement contributes to RSSC genetic diversity, we determined the positions of IS elements within RSSC isolates. IS were found to be broadly distributed across RSSC genomes although IS hotspots and coldspots were identified. IS hotspots have previously been identified in other bacterial species [68] and have been linked to a high density of IS recognition sites. However, IS cold spots may reflect either a lack of preferred IS insertion sites or selection against insertions due to high density of essential housekeeping genes [69]. Across RSSC phylotypes, IS genome positions were found to be strongly associated with host genetic diversity with genetically similar isolates having similar IS positions. This is a typical signature of vertical transmission from a common ancestor and suggests that insertion sequences could be used as molecular markers to track evolution within RSSC lineages [70]. The prevalence of unique insertion sites also varied between phylotypes; while phylotype I insertion sites were generally found in small numbers of isolates, phylotype IIB insertion sites were often shared by most isolates. Firstly, this suggests that that the high abundance of IS5 and IS3 families in the clonal phylotype IIB sub-lineage may be due to vertical transmission and clonal expansion rather than repeated unique insertion events. Further, it suggests that IS movement may be greater in some RSSC lineages than others hence potentially contributing to both intra- and inter-lineage genetic variation.

Insertions nearby or inside genes can result in both gene disruption [6, 52, 71, 72] and gene activation [73–75]. In RSSC, IS insertions have been found to disrupt type III effectors [26] and global virulence regulators [28]. We found that the majority of IS (71.4%) were inserted within 100 bp of their closest neighbouring gene’s start codon and could potentially have affected gene expression (although the positions of IS relative to gene promoters is unclear). Such potential IS-associated fitness effects should be addressed further by analysing gene promoter positions in both the bacterial genomes and in the IS to determine whether promoters are disrupted or modified. Only 4.9 % of IS were found to disrupt genes despite putative coding sequences comprising approximately 86.8 % of the RSSC genome [33]. Similarly, low proportions of IS-mediated gene disruptions have been found in other pathogens [55], suggesting there may be strong purifying selection against intra-genic insertions.

Most gene disruptions were found in small genetically similar clades in phylotype I, suggesting they likely represent recent insertions and may provide local adaptive benefits. IS gene disruptions in phylotype IIB also occurred in genetically similar clades but, due to the presence of a highly clonal IIB sub-lineage, were found in most isolates. Consistent with previous analyses [26], IS disruptions were found in various genes with potential roles in bacterial virulence, competition and stress tolerance, including type III effectors, membrane transporters, virulence regulators, and proteins involved in antibiotic and oxidative stress tolerance. Disruption of some of these genes, such as type III effectors, may be under strong selection given they have key roles in RSSC growth and infection [76]. However, other IS disruptions, particularly in phylotype IIB isolates, included integrases, MFS transporters, and AraC transcriptional regulators which have been shown to be preferential IS insertion sites across prokaryotic phyla [68]. The existence of preferential insertion sites in RSSC genomes may explain why some IS genome positions and gene disruptions are so conserved. They also make it difficult to predict the fitness effects of IS insertions given IS position conservation may be independent of selection. Therefore, further experimental analyses on the fitness impact of RSSC IS gene disruptions are required.

In conclusion, this study provides insights into the distribution and potential spread of IS across the RSSC. Our results highlight that although IS are widespread on the family level, they are lineage-specific on the subgroup level and are tightly bound to the evolution of their hosts. Further, while IS are broadly distributed in RSSC genomes, they cause gene disruptions within lineages that are shared by genetically similar isolates. Therefore, IS may affect host fitness both within lineages and across the whole RSSC.

## Supplementary Data

Supplementary material 1

Supplementary material 2

Supplementary material 3

Supplementary material 4

Supplementary material 5

## References

[1] Arber W (2000). Genetic variation: molecular mechanisms and impact on microbial evolution. FEMS Microbiol Rev.

[2] Che Y, Yang Y, Xu X, Břinda K, Polz MF (2021). Conjugative plasmids interact with insertion sequences to shape the horizontal transfer of antimicrobial resistance genes. Proc Natl Acad Sci.

[3] Mahillon J, Chandler M (1998). Insertion sequences. Microbiol Mol Biol Rev.

[4] Siguier P, Gourbeyre E, Chandler M (2014). Bacterial insertion sequences: their genomic impact and diversity. FEMS Microbiol Rev.

[5] Fowler RC, Hanson ND (2014). Emergence of carbapenem resistance due to the novel insertion sequence ISPa8 in *Pseudomonas aeruginosa*. PLoS One.

[6] Boutoille D, Corvec S, Caroff N, Giraudeau C, Espaze E (2004). Detection of an IS21 insertion sequence in the mexR gene of *Pseudomonas aeruginosa* increasing beta-lactam resistance. FEMS Microbiol Lett.

[7] Graves JL, Tajkarimi M, Cunningham Q, Campbell A, Nonga H (2015). Rapid evolution of silver nanoparticle resistance in *Escherichia coli*. Front Genet.

[8] Perez M, Calles-Enríquez M, del Rio B, Ladero V, Martín MC (2015). IS256 abolishes gelatinase activity and biofilm formation in a mutant of the nosocomial pathogen *Enterococcus faecalis* V583. Can J Microbiol.

[9] Garnier F, Janapatla RP, Charpentier E, Masson G, Grélaud C (2007). Insertion sequence 1515 in the ply gene of a type 1 clinical isolate of *Streptococcus pneumoniae* abolishes pneumolysin expression. J Clin Microbiol.

[10] Benson MA, Ohneck EA, Ryan C, Alonzo F, Smith H (2014). Evolution of hypervirulence by a MRSA clone through acquisition of a transposable element. Mol Microbiol.

[11] Rezwan F, Lan R, Reeves PR (2004). Molecular basis of the indole-negative reaction in *Shigella* strains: extensive damages to the tna operon by insertion sequences. J Bacteriol.

[12] Gaffé J, McKenzie C, Maharjan RP, Coursange E, Ferenci T (2011). Insertion sequence-driven evolution of *Escherichia coli* in chemostats. J Mol Evol.

[13] Lartigue MF, Poirel L, Nordmann P (2004). Diversity of genetic environment of *bla*(CTX-M) genes. FEMS Microbiol Lett.

[14] Lartigue MF, Poirel L, Aubert D, Nordmann P (2006). In vitro analysis of IS*Ecp1B*-mediated mobilization of naturally occurring beta-lactamase gene *bla*CTX-M of *Kluyvera ascorbata*. Antimicrob Agents Chemother.

[15] Cluzel PJ, Chopin A, Ehrlich SD, Chopin MC (1991). Phage abortive infection mechanism from *Lactococcus lactis* subsp. lactis, expression of which is mediated by an Iso-ISS1 element. Appl Environ Microbiol.

[16] Han HJ, Kuwae A, Abe A, Arakawa Y, Kamachi K (2011). Differential expression of type III effector BteA protein due to IS481 insertion in *Bordetella pertussis*. PLoS One.

[17] Wood MS, Byrne A, Lessie TG (1991). IS406 and IS407, two gene-activating insertion sequences for *Pseudomonas cepacia*. Gene.

[18] Bongers RS, Hoefnagel MHN, Starrenburg MJC, Siemerink MAJ, Arends JGA (2003). IS981-mediated adaptive evolution recovers lactate production by ldhB transcription activation in a lactate dehydrogenase-deficient strain of *Lactococcus lactis*. J Bacteriol.

[19] Vandecraen J, Chandler M, Aertsen A, Van Houdt R (2017). The impact of insertion sequences on bacterial genome plasticity and adaptability. Crit Rev Microbiol.

[20] Rajeshwari R, Sonti RV (2000). Stationary-phase variation due to transposition of novel insertion elements in *Xanthomonas oryzae* pv. oryzae. J Bacteriol.

[21] Chatterjee S, Sonti RV (2005). Virulence deficiency caused by a transposon insertion in the purH gene of *Xanthomonas oryzae* pv. *oryzae*. Can J Microbiol.

[22] González AI, Ruiz ML, Polanco C (1998). A race-specific insertion of transposable element IS801 in *Pseudomonas syringae* pv. *phaseolicola*. Mol Plant Microbe Interact.

[23] Noël L, Thieme F, Nennstiel D, Bonas U (2002). Two novel type III-secreted proteins of *Xanthomonas campestris* pv. *vesicatoria* are encoded within the hrp pathogenicity island. J Bacteriol.

[24] Hanekamp T, Kobayashi D, Hayes S, Stayton MM (1997). Avirulence gene D of *Pseudomonas syringae* pv. tomato may have undergone horizontal gene transfer. FEBS Lett.

[25] Kim JF, Charkowski AO, Alfano JR, Collmer A, Beer SV (1998). Sequences related to transposable elements and bacteriophages flank avirulence genes of *Pseudomonas syringae*. MPMI.

[26] Gonçalves OS, Campos KF, de Assis JCS, Fernandes AS, Souza TS (2020). Transposable elements contribute to the genome plasticity of *Ralstonia solanacearum* species complex. Microb Genom.

[27] Lavie M, Seunes B, Prior P, Boucher C (2004). Distribution and sequence analysis of a family of type ill-dependent effectors correlate with the phylogeny of *Ralstonia solanacearum* strains. Mol Plant Microbe Interact.

[28] Jeong EL, Timmis JN (2000). Novel insertion sequence elements associated with genetic heterogeneity and phenotype conversion in *Ralstonia solanacearum*. J Bacteriol.

[29] Alderley CL, Greenrod STE, Friman V-P (2022). Plant pathogenic bacterium can rapidly evolve tolerance to an antimicrobial plant allelochemical. Evol Appl.

[30] Gonçalves OS, Souza F de O, Bruckner FP, Santana MF, Alfenas-Zerbini P (2021). Widespread distribution of prophages signaling the potential for adaptability and pathogenicity evolution of *Ralstonia solanacearum* species complex. Genomics.

[31] Genin S (2010). Molecular traits controlling host range and adaptation to plants in *Ralstonia solanacearum*. New Phytol.

[32] Hayward AC (1991). Biology and epidemiology of bacterial wilt caused by *Pseudomonas solanacearum*. Annu Rev Phytopathol.

[33] Remenant B, Coupat-Goutaland B, Guidot A, Cellier G, Wicker E (2010). Genomes of three tomato pathogens within the *Ralstonia solanacearum* species complex reveal significant evolutionary divergence. BMC Genomics.

[34] (2005). Bacterial Wilt Disease and the Ralstonia Solanacearum Species Complex [Internet].

[35] Safni I, Cleenwerck I, De Vos P, Fegan M, Sly L (2014). Polyphasic taxonomic revision of the *Ralstonia solanacearum* species complex: proposal to emend the descriptions of *Ralstonia solanacearum* and *Ralstonia syzygii* and reclassify current *R. syzygii* strains as *Ralstonia syzygii* subsp. *syzygii* subsp. nov., *R. solanacearum* phylotype IV strains as *Ralstonia syzygii* subsp. *indonesiensis* subsp. nov., banana blood disease bacterium strains as *Ralstonia syzygii* subsp. *celebesensis* subsp. nov. and *R. solanacearum* phylotype I and III strains as *Ralstonia pseudosolanacearum* sp. nov. Int J Syst Evol Microbiol.

[36] Lowe-Power TM, Hendrich CG, von Roepenack-Lahaye E, Li B, Wu D (2018). Metabolomics of tomato xylem sap during bacterial wilt reveals *Ralstonia solanacearum* produces abundant putrescine, a metabolite that accelerates wilt disease. Environ Microbiol.

[37] Williamson L, Nakaho K, Hudelson B, Allen C (2002). *Ralstonia solanacearum* race 3, biovar 2 strains isolated from geranium are pathogenic on potato. Plant Dis.

[38] Colburn-Clifford JM, Scherf JM, Allen C (2010). *Ralstonia solanacearum* Dps contributes to oxidative stress tolerance and to colonization of and virulence on tomato plants. Appl Environ Microbiol.

[39] Tjou-Tam-Sin NNA, van de Bilt JLJ, Westenberg M, Gorkink-Smits PPMA, Landman NM (2017). Assessing the pathogenic ability of *Ralstonia pseudosolanacearum* (*Ralstonia solanacearum* phylotype I) from ornamental *Rosa* spp. plants. Front Plant Sci.

[40] Bocsanczy AM, Huguet-Tapia JC, Norman DJ (2017). Comparative genomics of *Ralstonia solanacearum* identifies candidate genes associated with cool virulence. Front Plant Sci.

[41] Gonçalves OS, de Queiroz MV, Santana MF (2020). Potential evolutionary impact of integrative and conjugative elements (ICEs) and genomic islands in the *Ralstonia solanacearum* species complex. Sci Rep.

[42] Greenrod STE, Stoycheva M, Elphinstone J, Friman VP (2022). Global diversity and distribution of prophages are lineage-specific within the *Ralstonia solanacearum* species complex. BMC Genomics.

[43] Xie Z, Tang H (2017). ISEScan: automated identification of insertion sequence elements in prokaryotic genomes. Bioinformatics.

[44] Hawkey J, Hamidian M, Wick RR, Edwards DJ, Billman-Jacobe H (2015). ISMapper: identifying transposase insertion sites in bacterial genomes from short read sequence data. BMC Genomics.

[45] Wick RR, Judd LM, Gorrie CL, Holt KE (2017). Unicycler: resolving bacterial genome assemblies from short and long sequencing reads. PLoS Comput Biol.

[46] Ondov BD, Treangen TJ, Melsted P, Mallonee AB, Bergman NH (2016). Mash: fast genome and metagenome distance estimation using MinHash. Genome Biol.

[47] Li H, Durbin R (2009). Fast and accurate short read alignment with Burrows-Wheeler transform. Bioinformatics.

[48] Li H, Handsaker B, Wysoker A, Fennell T, Ruan J (2009). The sequence alignment/map format and SAMtools. Bioinformatics.

[49] Paradis E, Claude J, Strimmer K (2004). APE: Analyses of Phylogenetics and Evolution in R language. Bioinformatics.

[50] Hutchinson MC, Cagua EF, Balbuena JA, Stouffer DB, Poisot T (2017). paco: implementing Procrustean Approach to Cophylogeny in R. Methods Ecol Evol.

[51] Yu G, Smith DK, Zhu H, Guan Y, Lam TTY (2017). Ggtree: an R package for visualization and annotation of phylogenetic trees with their covariates and other associated data. Methods Ecol Evol.

[52] Moffatt JH, Harper M, Adler B, Nation RL, Li J (2011). Insertion sequence ISAba11 is involved in colistin resistance and loss of lipopolysaccharide in *Acinetobacter baumannii*. Antimicrob Agents Chemother.

[53] Simser JA, Rahman MS, Dreher-Lesnick SM, Azad AF (2005). A novel and naturally occurring transposon, ISRpe1 in the Rickettsia peacockii genome disrupting the rickA gene involved in actin-based motility. Mol Microbiol.

[54] De Palmenaer D, Siguier P, Mahillon J (2008). IS4 family goes genomic. BMC Evol Biol.

[55] Hawkey J, Monk JM, Billman-Jacobe H, Palsson B, Holt KE (2020). Impact of insertion sequences on convergent evolution of *Shigella* species. PLoS Genet.

[56] Consuegra J, Gaffé J, Lenski RE, Hindré T, Barrick JE (2021). Insertion-sequence-mediated mutations both promote and constrain evolvability during a long-term experiment with bacteria. Nat Commun.

[57] Genin S, Boucher C (2004). Lessons learned from the genome analysis of ralstonia solanacearum. Annu Rev Phytopathol.

[58] Craig NL (1997). Target site selection in transposition. Annu Rev Biochem.

[59] Hu WY, Derbyshire KM (1998). Target choice and orientation preference of the insertion sequence IS903. J Bacteriol.

[60] Ton-Hoang B, Pasternak C, Siguier P, Guynet C, Hickman AB (2010). Single-stranded DNA transposition is coupled to host replication. Cell.

[61] Guérillot R, Da Cunha V, Sauvage E, Bouchier C, Glaser P (2013). Modular evolution of TnGBSs, a new family of integrative and conjugative elements associating insertion sequence transposition, plasmid replication, and conjugation for their spreading. J Bacteriol.

[62] Brochet M, Da Cunha V, Couvé E, Rusniok C, Trieu-Cuot P (2009). Atypical association of DDE transposition with conjugation specifies a new family of mobile elements. Mol Microbiol.

[63] Ramos-González MI, Campos MJ, Ramos JL, Espinosa-Urgel M (2006). Characterization of the *Pseudomonas putida* mobile genetic element ISPpu10: an occupant of repetitive extragenic palindromic sequences. J Bacteriol.

[64] Clément JM, Wilde C, Bachellier S, Lambert P, Hofnung M (1999). IS1397 is active for transposition into the chromosome of *Escherichia coli* K-12 and inserts specifically into palindromic units of bacterial interspersed mosaic elements. J Bacteriol.

[65] Tobes R, Pareja E (2006). Bacterial repetitive extragenic palindromic sequences are DNA targets for Insertion sequence elements. BMC Genomics.

[66] Leclercq S, Cordaux R (2011). Do phages efficiently shuttle transposable elements among prokaryotes?. Evolution.

[67] Oliveira PH, Touchon M, Cury J, Rocha EPC (2017). The chromosomal organization of horizontal gene transfer in bacteria. Nat Commun.

[68] Tempel S, Bedo J, Talla E (2022). From a large-scale genomic analysis of insertion sequences to insights into their regulatory roles in prokaryotes. BMC Genomics.

[69] van Opijnen T, Levin HL (2020). Transposon insertion sequencing, a global measure of gene function. Annu Rev Genet.

[70] Stanley J, Saunders N (1996). DNA insertion sequences and the molecular epidemiology of *Salmonella* and *Mycobacterium*. J Med Microbiol.

[71] Hernández-Allés S, Benedí VJ, Martínez-Martínez L, Pascual A, Aguilar A (1999). Development of resistance during antimicrobial therapy caused by insertion sequence interruption of porin genes. Antimicrob Agents Chemother.

[72] Kobayashi K, Tsukagoshi N, Aono R (2001). Suppression of hypersensitivity of *Escherichia coli* acrB mutant to organic solvents by integrational activation of the acrEF operon with the IS1 or IS2 element. J Bacteriol.

[73] Wachino J, Yamane K, Kimura K, Shibata N, Suzuki S (2006). Mode of transposition and expression of 16S rRNA methyltransferase gene rmtC accompanied by ISEcp1. Antimicrob Agents Chemother.

[74] Blount ZD, Barrick JE, Davidson CJ, Lenski RE (2012). Genomic analysis of a key innovation in an experimental *Escherichia coli* population. Nature.

[75] Sóki J, Gal M, Brazier JS, Rotimi VO, Urbán E (2006). Molecular investigation of genetic elements contributing to metronidazole resistance in *Bacteroides* strains. J Antimicrob Chemother.

[76] Lei N, Chen L, Kiba A, Hikichi Y, Zhang Y (2020). Super-multiple deletion analysis of type III effectors in *Ralstonia solanacearum* OE1-1 for full virulence toward host plants. Front Microbiol.

