# Supplementary material for: Influence of insertion sequences on population structure of phytopathogenic bacteria in the Ralstonia solanacearum species complex
Source: Microbiology (Reading). 2023 Jul 17;169(7):001364. doi: 10.1099/mic.0.001364 (PMC10433421; doi:10.1099/mic.0.001364)
Supplement: Supplementary material 1 [file mic-169-1364-s001.pdf]

Influence of insertion sequences on population structure of phytopathogenic bacteria in the *Ralstonia solanacearum* species complex

Authors: Samuel TE Greenrod*, Martina Stoycheva, John Elphinstone, Ville-Petri Friman*

Supplementary figures

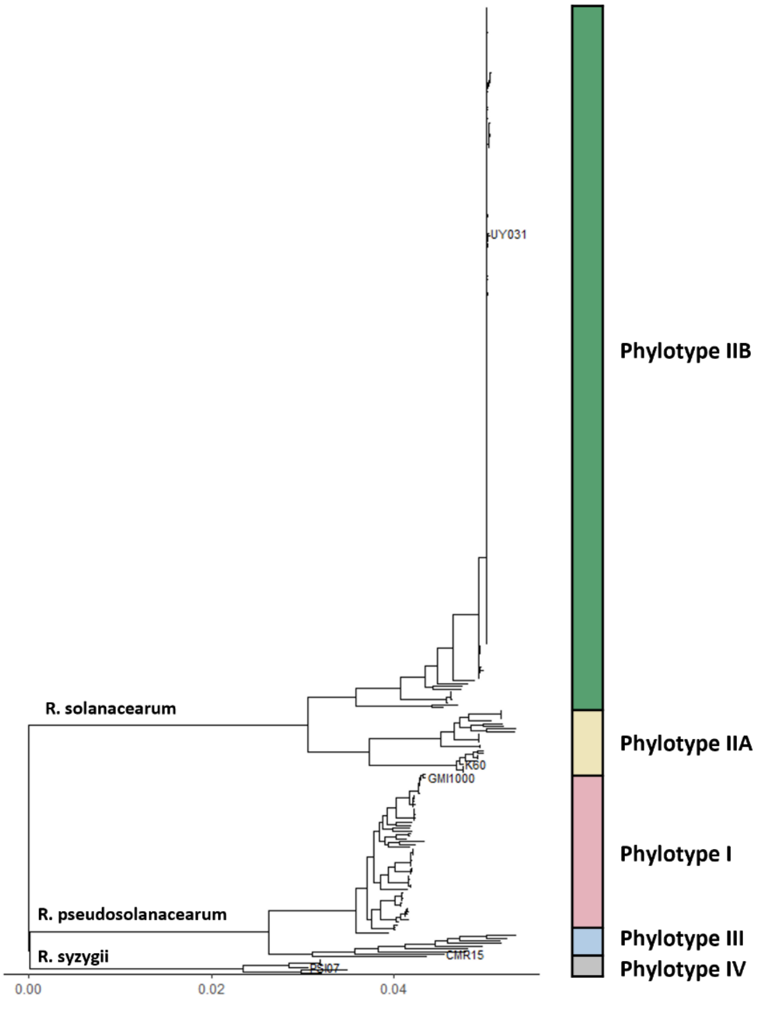

Figure S1. Phylogeny of *Ralstonia solanacearum* species complex. Maximum Likelihood phylogeny was constructed based on the genomes of 356 *Ralstonia solanacearum* species complex strains the National Collection of Plant Pathogenic Bacteria (NCPPB) and other reference strains maintained at Fera Science Ltd, along with 5 previously phylotyped and sequenced strains from NCBI Genbank (names shown at the tips of tree). Phylogenetic relationships between known phylotypes were used to assign the 356 strains sequenced in this study to given phylotype clusters (coloured bar) and species (names shown on tree branches). Scale bar (nucleotide substitutions per site) is provided.

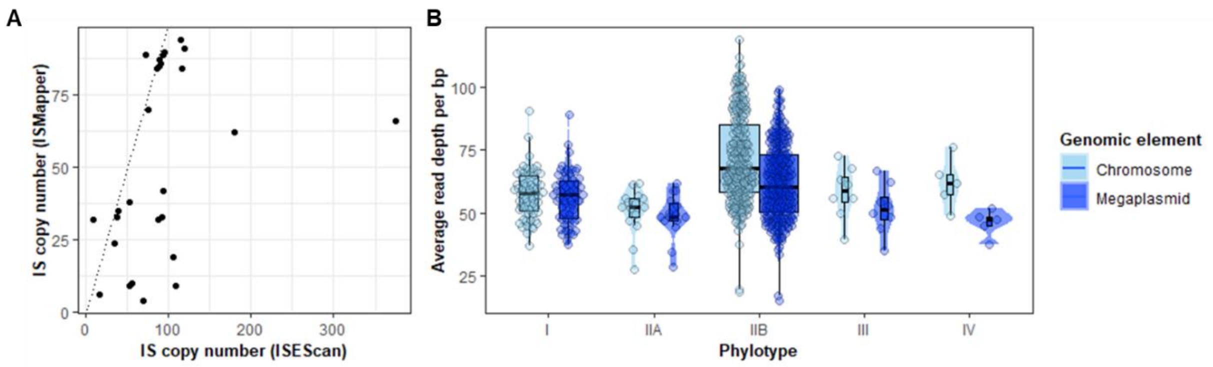

Figure S2. Short read IS detection is correlated with long read assemblies and is unaffected by average read depth. A) Scatterplot showing IS copy number determined using ISEScan (long read assemblies) against ISMapper (short read data). Dotted line has slope = 1, intercept = 0 and shows the expected relationship if both methods find the same copy number. B) Boxplot and violin plot showing average read depth per isolate for each phylotype. Data is scattered to reduce overplotting. The difference in read depth between phylotypes was tested statistically using a Kruskal-Wallis test.

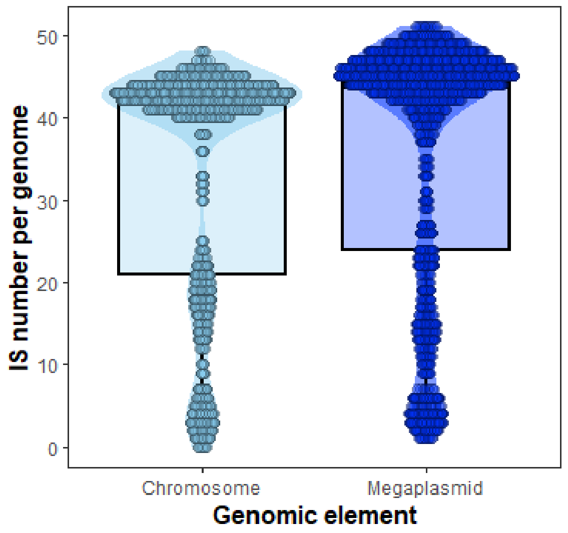

Figure S3. The megaplasmid contains significantly more IS than the chromosome. Boxplot showing IS copy number in the chromosome and the megaplasmid. Boxplots are coloured by genomic region. The difference in IS number between regions was tested statistically using a paired t-test.

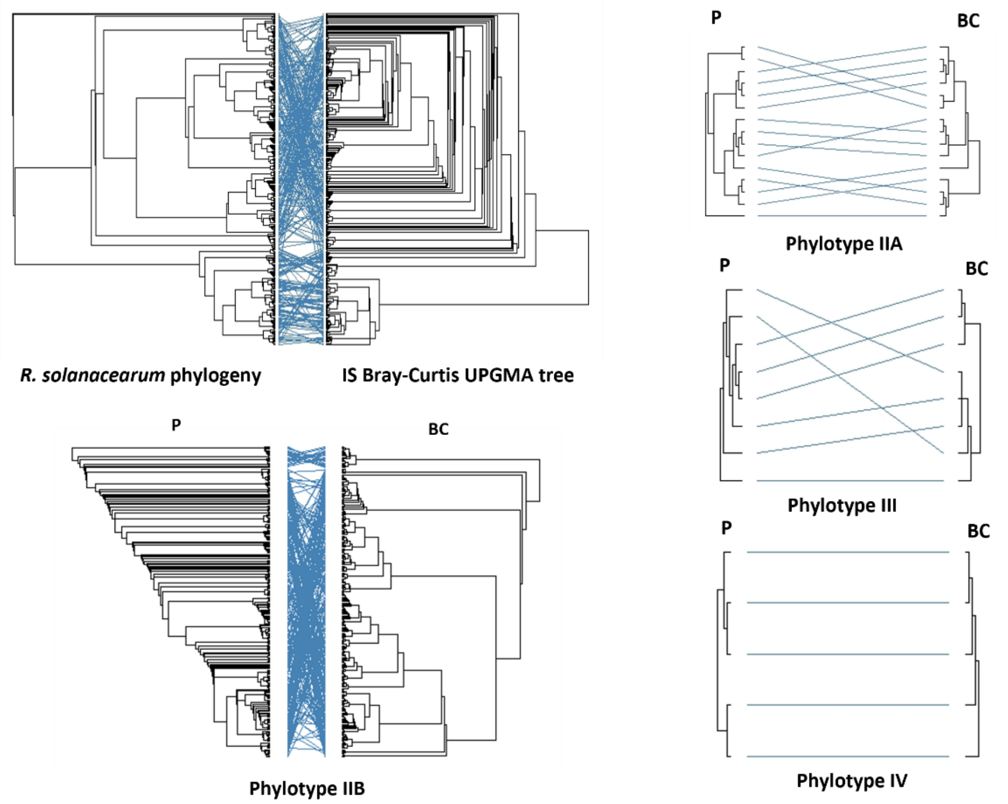

Figure S4. IS subgroups composition is closely associated with host phylogenetic similarity. Tanglegrams showing the congruence between RSSC phylogeny (left side) and UPGMA tree (right side) calculated using Bray-Curtis dissimilarity of IS presence. The first tanglegram shows congruence for the whole phylogeny and the other plots show congruence within lineages. Phylogeny is labelled with P and UPGMA tree is labelled with BC (Bray-Curtis).

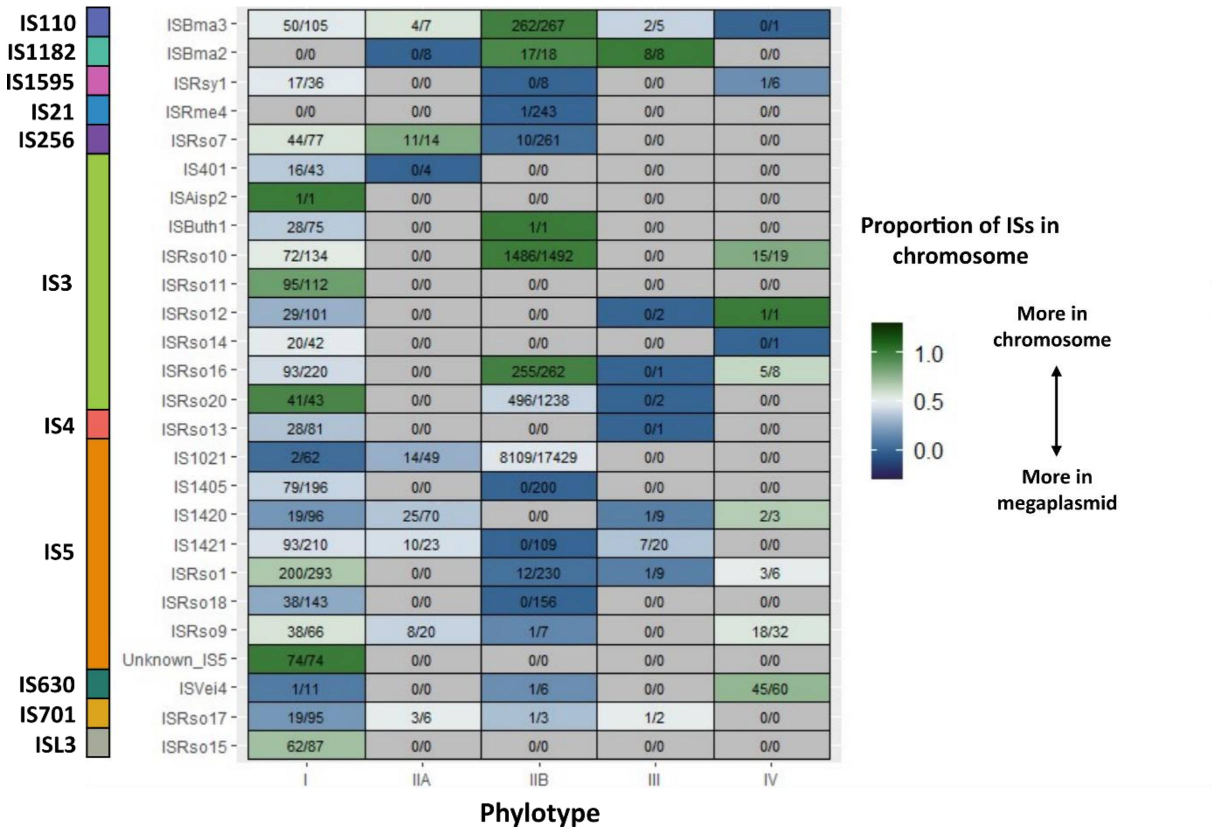

Figure S5. IS subgroups are located in different genomic regions in different lineages. Heatmap showing the proportion of each IS subgroup found in the chromosome in each host lineage (IS subgroup copies in chromosome/total IS subgroup copies for each lineage). Fractions are shown in boxes. Cells with a high proportion of chromosomal IS are shown in green and cells with a high proportion of megaplasmid IS are shown in blue. Grey cells are where an IS subgroup was not present in the lineage. IS are clustered by IS family as shown by the coloured bar and IS family labels.

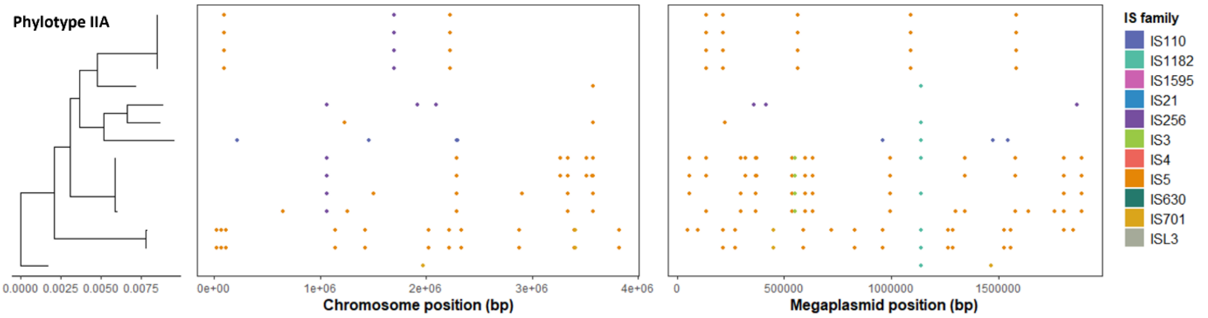

Figure S6. IS are highly mobile in RSSC genomes but IS positions show strong association with bacterial phylogenetic similarity. Dot plots showing the distribution of IS in both the chromosome and megaplasmid across phylotype IIA strains. Each dot represents a separate IS position is coloured by IS family. Scale bar (nucleotide substitutions per site) is shown for phylogeny.

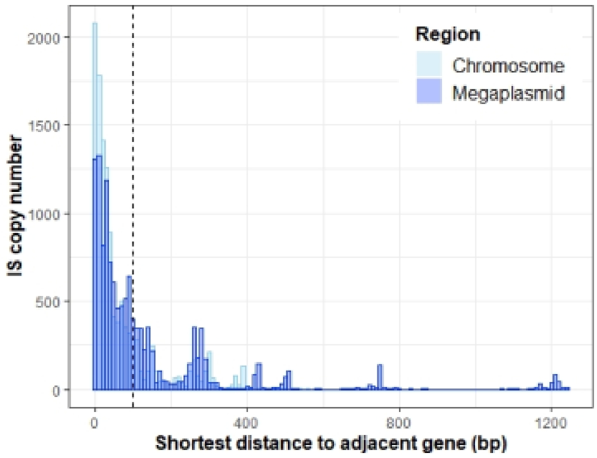

Figure S7. IS are situated close to neighbouring genes. Histogram of the shortest predicted distance between each IS and its adjacent gene's start codon. Histograms for chromosomal and megaplasmid IS are overlaid and coloured separately. Dotted vertical line shows 100 bp threshold.

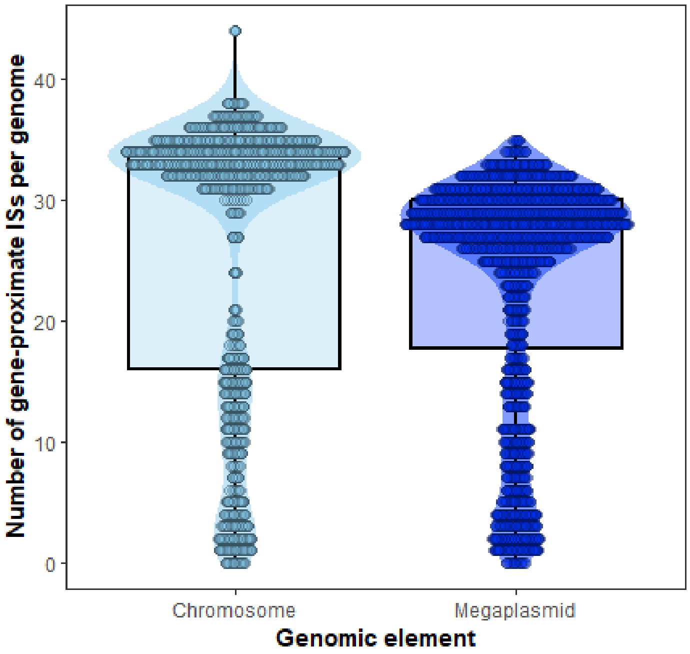

Figure S8. The megaplasmid contains significantly fewer gene-proximate IS than the chromosome. Boxplot showing number of IS that are close to (< 100 bp from start codon) or disrupt genes in the chromosome and the megaplasmid. Boxplots are coloured by genomic region. The difference in IS number between regions was tested statistically using a paired t-test.

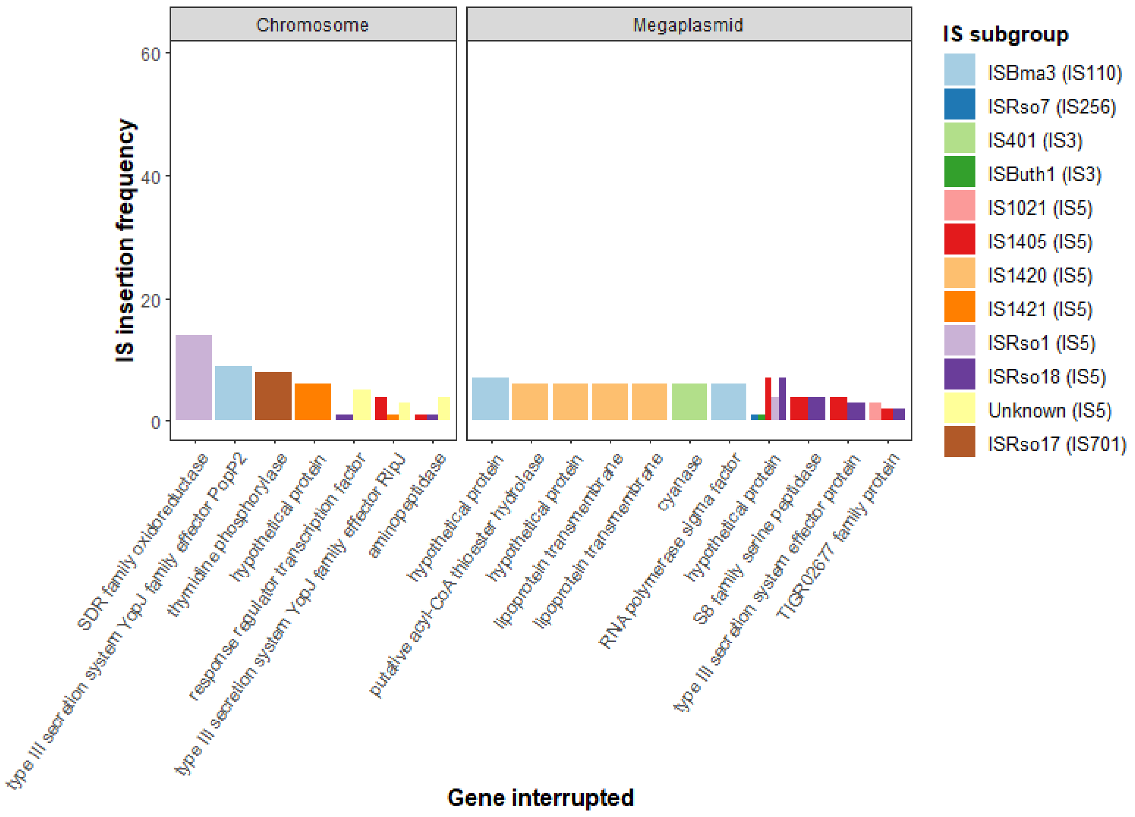

Figure S9. Phylotype I IS gene disruptions are caused by multiple IS subgroups. Bar plots showing the prevalence and functional annotations of unique disrupted genes in phylotype I isolates. Only genes that contained disruptions in five or more isolates are shown. Genes are ordered by frequency of IS disruption from left to right. Dot plots and bar plots are coloured by IS subgroup (IS family in brackets).
